# Inferring Cell-Scale Signalling Networks via Compressive Sensing

**DOI:** 10.1371/journal.pone.0095326

**Published:** 2014-04-18

**Authors:** Lei Nie, Xian Yang, Ian Adcock, Zhiwei Xu, Yike Guo

**Affiliations:** 1 Department of Computing, Imperial College London, London, United Kingdom; 2 Institute of Computing Technology, Chinese Academy of Sciences, Beijing, China; 3 University of Chinese Academy of Sciences, Beijing, China; 4 National Heart and Lung Institute, Imperial College London, London, United Kingdom; Leibniz-Institute for Farm Animal Biology (FBN), Germany

## Abstract

Signalling network inference is a central problem in system biology. Previous studies investigate this problem by independently inferring local signalling networks and then linking them together via crosstalk. Since a cellular signalling system is in fact indivisible, this reductionistic approach may have an impact on the accuracy of the inference results. Preferably, a cell-scale signalling network should be inferred as a whole. However, the holistic approach suffers from three practical issues: scalability, measurement and overfitting. Here we make this approach feasible based on two key observations: 1) variations of concentrations are sparse due to separations of timescales; 2) several species can be measured together using cross-reactivity. We propose a method, CCELL, for cell-scale signalling network inference from time series generated by immunoprecipitation using Bayesian compressive sensing. A set of benchmark networks with varying numbers of time-variant species is used to demonstrate the effectiveness of our method. Instead of exhaustively measuring all individual species, high accuracy is achieved from relatively few measurements.

## Introduction

Inferring signalling networks from time series aims at revealing the mechanisms behind biological processes and is an important research subject in systems biology. Many local signalling networks (e.g. [1]–[3]) have been inferred from the dynamic concentrations of proteins typically quantified by immunoprecipitation [4]. Studies for inferring local signalling networks are based on the assumption that the target network is isolated from other networks in a cellular system. In most cells, at least one species in a local signalling network will have effects on other networks; that is known as crosstalk. For example, the glucocorticoid receptor (GR) pathway is vital for regulating anti-inflammatory and immunosuppressive processes. Post-translational modification of GR, a potential substrate for p38 mitogen-activated protein kinase (MAPK) pathway, affects nuclear retention of GR as well as transactivation. In some inflammatory diseases, such as severe asthma, the effect of GR as an anti-inflammatory regulator is dramatically impaired when the p38 MAPK is over-activated. This suggests that this is crosstalk between the p38 MAPK and GR pathways, which can potentially explain the reduced responsiveness to glucocorticoids in chronic inflammation at the molecular level. Although recent studies (e.g. [5]–[9]) have explored crosstalk and linked local signalling networks together, their approach still artificially divides the whole signalling system into many small-scale subsystems. Since a cellular signalling system is in fact indivisible, such reductionistic approach may have an impact on the accuracy of the inference results. An alternative approach is to infer a cell-scale signalling network without separation. This network captures the emergent properties of a whole-cell signal transduction system. In theory, a cell-scale signalling network can be inferred using existing methods, such as maximum likelihood estimation [2], least-squares estimation [10], [11], non-linear optimization [12], Kalman filters [13], [14] and approximate Bayesian computation [15], [16]. However, this holistic approach suffers from three practical issues, which limits the applications of the existing methods:

- *The scalability issue.* A cell-scale signalling network includes a huge number of proteins and their various forms. For instance, there are 518 kinases [17] and approximately 150 phosphatases [18] that together mediate the signalling network in a human cell. Exhaustively measuring all the proteins in a cell-scale signalling network via immunoprecipitation is extremely expensive and frequently impossible. Moreover, unlike regulatory network inference, in which gene expression levels can be measured by high-throughput technologies (e.g., microarray), it is very challenging to precisely quantify a large number of proteins and especially their post-translational modifications [19]. Although the emerging mass spectrometry technique can be successfully used to qualify proteomes [20], measuring post-translational modified proteins in signalling networks is highly dependent on enrichment methods whose performance is influenced by various factors [21].
- *The measurement issue.* It is impractical to individually measure all proteins via immunoprecipitation in a cell-scale signalling network due to their various post-translational modifications and complex formations. For example, in the JAK-STAT signalling pathway, unphosphorylated STAT5, tyrosine phosphorylated monomeric STAT5 and tyrosine phosphorylated dimeric STAT5 are difficult to assess individually [21].
- *The overfitting issue.* Few studies have attempted to provide cell-scale signalling networks, and as a result, little is known of their structure. It has been reported that the existing inference methods are likely to overfit for experimental data without structural constraints [22].

As a result, the methodology of inferring cell-scale signalling networks requires fundamental changes. This paper proposes a new method, called CCELL, that responds to all the three challenging issues described above and flows from the following two key observations:

- *Variations of concentrations are sparse due to separations of timescales.* The cell-scale signalling networks incorporate biological processes occur over different timescales. Typically, the receptor internalization (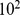
s) process triggers phosphorylation and catalysis of proteins (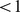
s) that in turn translocate into cell nucleus and induce their target gene expression; the transcriptional regulation process (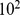
s), acting as a linkage point, stimulates signal cascading of other signalling pathways [23]. As a result, the concentrations of only a few species in a cell vary significantly at a specific timescale while the concentrations of a large fraction of species remain stable [24], [25]. This is because the processes over faster timescales reach their steady states instantaneously and the dynamics of the processes over slower timescales can be reasonably ignored. Thus a large number of variations of concentrations are zero or close to zero under a specific timescale, if we define the variations of concentrations as the differences between concentrations of adjacent time points. In other words, variations of concentrations are sparse.
- *Combined-measurements can be implemented using cross-reactivity.* Due to the cross-reactivity of an antibody, the antibody may bind not only the targeted protein but also other proteins, such as the various molecular forms of the target protein or other proteins in complex with the target protein [26]. This phenomenon frequently affects measurements of the concentration of the target protein in an immunoprecipitation assay. The traditional way is to use an antibody with a high specific affinity under stringent binding conditions in order to obtain accurate results. In contrast to the traditional way, we attempt to use the cross-reactivity of antibodies in order to measure the aggregated concentrations of several proteins in one go. We call this experimental method combined-measurement.

These two key observations motivated us to use compressive sensing as the foundation of our inference method for cell-scale signalling networks. Compressive sensing [27]–[29] is a revolutionary technique for signal reconstruction that uses a sampling rate far lower than the Nyquist-Shannon rate. Assuming that the signal of interest can be represented using a vector, compressive sensing requires that one measurement can acquire an inner product of the signal vector and a predefined measurement vector (i.e. a weighted sum of several predefined elements of the signal vector). All measurement vectors constitute a measurement matrix, while all results of measurements form an observation vector. Recovering the signal from an observation vector is a highly undetermined problem since the number of measurements is typically far lower than the number of elements of the signal vector. Compressive sensing can recover the signal by adding sparse constraints on the signal vector on the condition that the measurement matrix meets a prerequisite called restricted isometry property. Another approach is to use Bayesian compressive sensing that is a probabilistic version of compressive sensing [30], [31]. The primary advantage of Bayesian compressive sensing is that it does not require the measurement matrix to obey the restricted isometry property, but infers a distribution of the signal vector.

To sum up, Bayesian compressive sensing is based on the following two essential conditions: (I) the signal is sparse in some domain; (II) one measurement can obtain a weighted sum of several elements of the signal vector. Sparse variations and combined-measurements exactly meet these two prerequisites; therefore, Bayesian compressive sensing is a promising technique that can be adapted to infer cell-scale signalling networks from relatively few measurements. Moreover, it avoids measuring proteins individually and uses sparse constraints to prevent the estimated network model from overfitting for the observed data.

Our method, CCELL, is based on Bayesian compressive sensing, aiming at inferring cell-scale signalling networks as a whole from time series data generated by immunoprecipitation assays. In this paper, CCELL is applied to biological networks approximated by linear models. A set of benchmark networks with varying numbers of time-variant species is designed to demonstrate our method. These networks are derived from four well-studied signalling pathways: JAK-STAT, GR, ERK and p38, as well as crosstalk amongst them. Experimental results show that CCELL is effective for inferring benchmark networks without structure constraints. Instead of exhaustively measuring all individual species, high accuracy can be achieved from relatively few measurements.

## Methods

**Figure 1:**
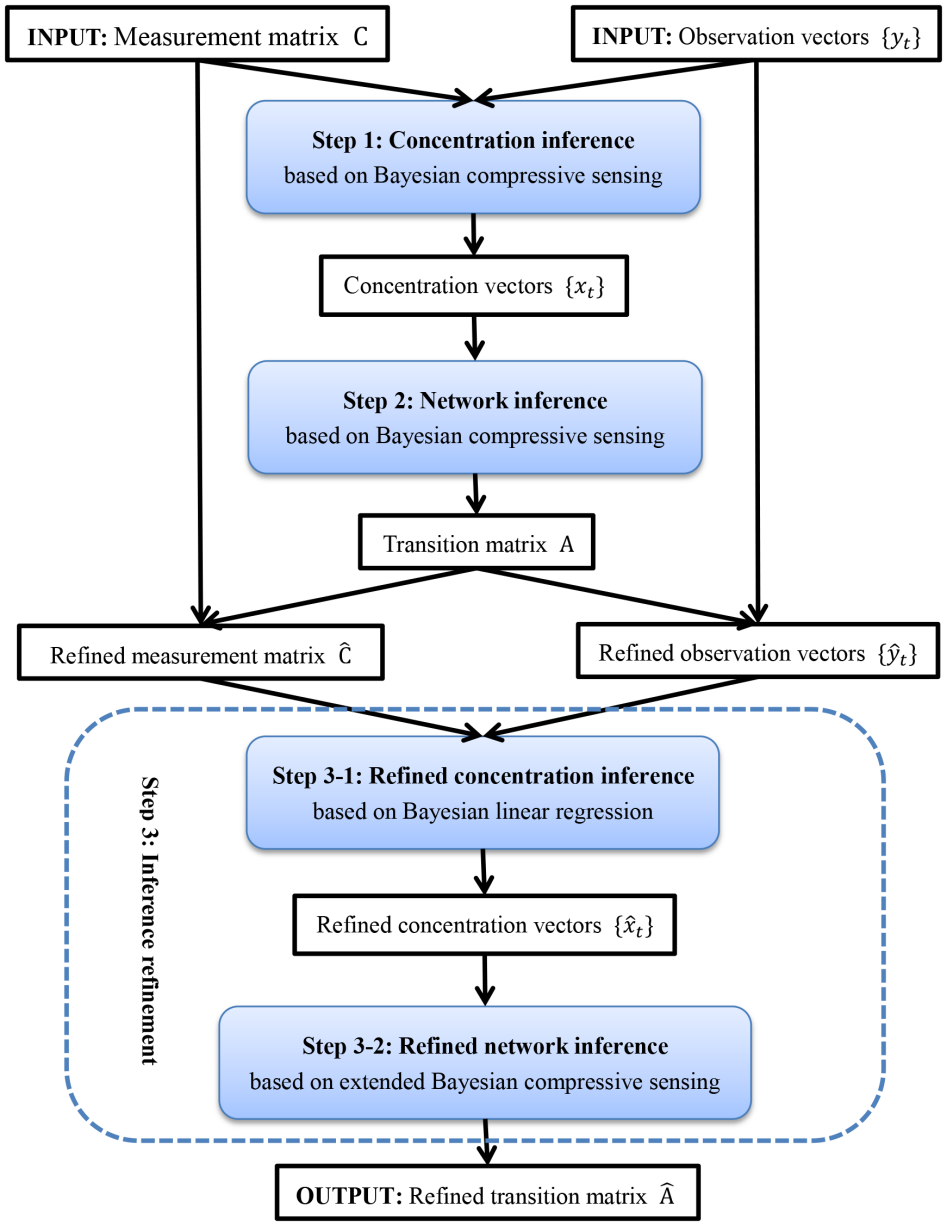
The workflow of CCELL. The CCELL method consists of 3 steps: concentration inference, network inference and inference refinement (including refined concentration inference and refined network inference). The core algorithm of the first two steps is Bayesian compressive sensing. The two substeps in Step 3 are based on Bayesian linear regression and extended Bayesian compressive sensing respectively. The input of CCELL is a measurement matrix 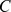 and its corresponding observation vectors 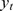 that are time-series generated by immunoprecipitation assays. The output of CCELL is a refined transition matrix 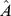 representing the cell-scale signalling network.

In this section, the core algorithm of CCELL, Bayesian compressive sensing, is first introduced. Then, we will explain the three sequential steps of CCELL: concentration inference, network inference and inference refinement. The structure of CCELL is detailed in Figure 1.

### Bayesian compressive sensing

Bayesian compressive sensing, introduced by Ji, Xue and Carin [31], is a probabilistic version of compressive sensing based on the relevance vector machine [30]. Let 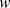 be the signal of interest that is represented using a 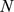-dimensional column vector. The sparsity of a vector is the proportion of (approximate) zero elements. A vector is sparse if its sparsity is greater than a threshold (usually 80%). A measurement matrix 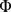 is a 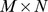-dimensional matrix, where 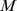 is the number of measurements. Typically, 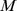 is far less than 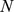. Each row of 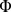 is a measurement vector, which is a 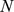-dimensional row vector. A measurement is to obtain the inner product of the signal vector and a measurement vector. For example, a measurement vector (i.e. a row of the measurement matrix) is 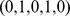 and the signal vector is 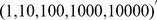. The prime symbol 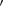 means the transpose of a vector or a matrix. The result of this measurement is the sum of the second and fourth elements, which is 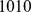. An observation vector 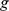 is 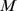-dimensional column vector, each element of which represents a measurement result of the corresponding measurement vector. Assuming the measurement noises are independent additive white Gaussian with mean 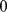 and the covariance matrix 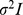, we can get a system of linear equations as follows: 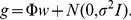
(1)The symbol 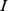 denotes an identity matrix. For simplicity, the dimension of 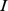 is various according to different equations without notation in this paper. Equation 1 is usually underdetermined, because the number of measurements 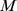 is far less than the number of elements of the signal vector 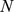. However, the additional assumption that the signal 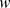 is sparse makes Equation 1 solvable. Bayesian compressive sensing is an inference algorithm to solve Equation 1 using a sparse prior distribution, which is typically Student's t-distribution. Its input is an observation vectors 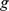 and a measurement matrix 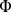. The corresponding output is a distribution of the signal 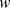.

Bayesian compressive sensing is an EM style iterative algorithm. Given a hyperparameter vector 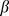 of the signal 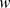, the E-step is to infer a posterior distribution of the signal 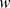. The posterior is a multivariate Gaussian distribution with the mean vector 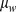 and the covariance matrix 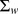 as follows: 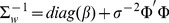
(2)

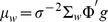
(3)where 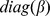 represents a diagonal matrix whose diagonal is the hyperparameter vector 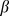. The M-step, based on the variational method[32], is to calculate an approximately optimal hyperparameters vector 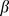 using the posterior of 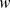 calculated in the previous E-step as follows:
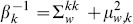
(4)where 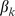 and 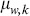 denote the 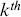 element of the hyperparameter vector 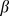 and the mean vector 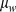 respectively; 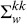 represents the element in the 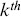 row and 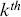 column of the covariance matrix 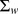.

Before the execution of the Bayesian compressive sensing algorithm, the hyperparameter vector is often set to a random or given value. Then, a posterior distribution of the signal is inferred by the E-step. Subsequently, the M-step update the hyperparameter vector based on the mean vector and the covariance matrix of the posterior distribution inferred in the previous E-step. Afterwards, the updated hyperparameter vector is used to infer a new posterior distribution in the E-step of the next iteration. The Bayesian compressive sensing algorithm iteratively executes the E-step and M-step until stop conditions are satisfied.

According to the workflow in Figure 1, the Bayesian compressive sensing algorithm is used in the concentration inference and network inference steps. This is because that both of the two steps aiming at solving systems of linear equations with sparse constraints, which have identical forms with Equation 1. Bayesian compressive sensing can directly solve these systems of linear equations. More specifically, in Figure 1 the concentration vector 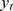, the measurement matrix 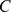 and the output concentration vector 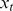 in Step 1 correspond to the observation vector 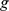, the measurement matrix 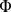 and the signal vector 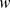 in Equation 1 respectively. Similarly, in Step 2 concentration vectors 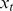 and 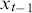 at two consecutive time points correspond to the observation vector 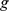 and the measurement matrix 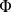, while the transition matrix 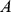 refers to the signal vector 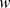 in Equation 1.

### Step 1: Concentration inference

Mathematically, combined-measurements are modelled as a system of linear equations: 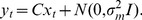
(5)

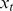
 is a concentration vector. Each element of 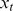 represents the concentration of a species at time 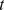, which is an unknown variable to be inferred. The dimension of 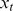 equals to the number of species in the network, denoted as 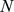. 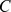 is a measurement matrix that is given in advance. Each row of 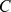 represents a combined-measurement. The dimension of 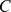 is 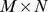, where 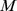 is the number of measurements and 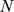 is the number of species. 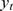 is an observation vector. Each element of 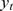 represents the observed value of a measurement at time 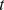. The dimension of 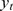 is the number of measurements 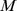. The random vector 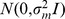 is measurement noises with mean 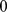 and the covariance matrix 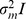. The variation of concentrations 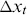 is defined as the difference between the concentration vectors at two adjacent time points:
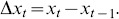
(6)Similarly, the variation of observations is defined as the difference between observation vectors at two adjacent time points:



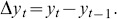
(7)The sparsity of variations is defined as the ratio between the number of time-invariant species and the number of all species. This definition is consistent with the definition of sparsity for a vector. According to the observation that the concentrations of only a few species in a cellular system vary significantly over a specific timescale, variations of concentrations are sparse. Therefore, Bayesian compressive sensing can be used to infer variations of concentrations by solving the following system of linear equations: 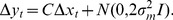
(8)


In wet lab experiments, a cell is perturbed from its steady state by triggers. As a large fraction of species at steady state have zero concentrations [3], the initial concentrations of all species can be inferred by Bayesian compressive sensing directly. Therefore, it is assumed that initial concentrations are known in this paper. Concentration vector 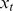 at other time points can be calculated according to Equation 6.

### Step 2: Network inference

This paper focuses on the biological networks that can be modelled by a system of linear equations: 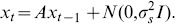
(9)

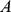
 is a transition matrix, whose elements are unknown variables to be inferred. The dimension of 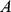 is 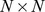, where 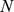 denotes the number of species. 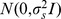 is system noises with mean 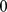 and covariance matrix 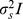. The networks modelled by differential equations can be also approximated by linear equations. One method is to define the transition matrix as a function of time, which can be calculated according to Jacobian matrices of the transition function [10]. The other method is to view higher order derivatives of concentrations as first order variables [22].

According to Equation 9, the 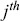 row of 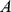, 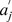, satisfies the following equation: 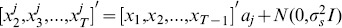
(10)where 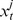 denotes the 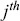 element of concentration vector 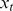. Equation 10 is only for the time-series profile of species under one perturbation. It can be easily extended to any number of perturbations by successively combining all profiles together. Equation 10 can fit the form of Equation 1. The transpose of the 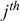 row 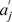 is the signal to be inferred. The matrix 
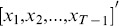
 and column vector 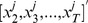 can be viewed as a measurement matrix and the corresponding observation vector respectively. According to a widely accepted assumption that structures of biological networks are usually sparse [3], [33], [34], Bayesian compressive sensing can be directly used to solve Equation 10. Thus, a posterior of the 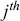 row of the transition matrix 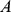 is calculated. Other rows can be independently inferred in a same way.

### Step 3: Inference refinement

#### Structural indicator

**Figure 2:**
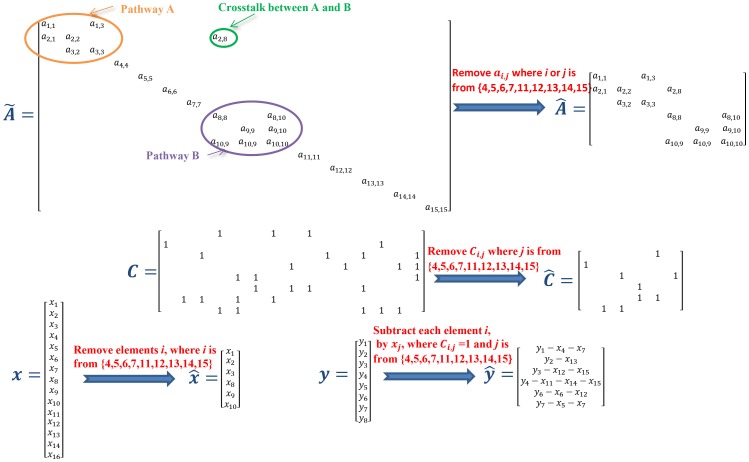
The process of refinement. The equations at the left and right side of arrows are original and reduced respectively. According to inferred transition matrix 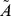, there are two pathway and crosstalk between them. The 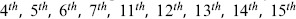 species are silent, having no links with others. All elements associated with the silent species are removed from the transition matrix 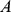 to form the refined transition matrix 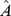. All columns measuring the silent species are deleted from the measurement matrix 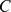 to form the refined measurement matrix 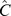. The refined concentration vector, 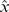, only keeps the concentrations of active species (e.g., 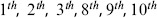). An element of the refined observation vector 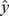 is equal to the corresponding element of the observation vector 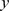 subtracted by the concentrations of the silent species involved in this measurement. If all species involved in a measurement are silent, simply remove this measurement.

For an inferred transition matrix 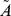 outputted by Step 2, the structural indicator is defined as follows: 
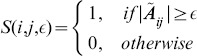
(11)where 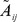 represents the inferred value of the element in 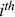 row and 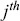 column of matrix 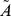; 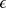 is a threshold parameter. If 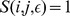, there is a link from species 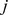 to 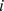 over the predetermined timescale of experiments. If a species has no links with other species, it is called as a silent species over the timescale; otherwise, it is called as an active species. It is noteworthy that a time-invariant species can be an active species, such as an enzyme that catalyses other species without changing its concentration. The process of refinement is to remove silent species in order to formulate a small scale inference problem, which is detailed in Figure 2.

#### Refined concentration inference

All silent species over the predetermined timescale are removed. The refined concentration vector, 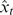, only contains the concentrations of active species. Each element of 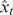 represents the concentration of an active species at time 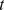, which is an unknown variable to be inferred. The refined measurement matrix 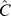 is derived from 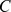 by removing all columns associated with silent species. An element of the refined measurements 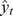 are calculated by subtracting concentrations of silent species involved in this measurement. Thus, the refined measurement model is as follows: 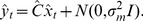
(12)


It is noteworthy that the variations of 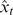 are not sparse. The assumptions of Bayesian compressive sensing are not satisfied. Instead, Bayesian linear regression is used to infer the posterior distribution of 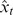. The posterior is a multivariate Gaussian distribution with the mean vector 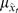 and the covariance matrix 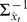 as follows: 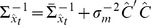
(13)

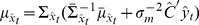
(14)where 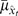 and 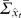 are the mean vector and the covariance matrix of the prior distribution of 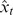 respectively. The prior distribution of 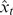 can be calculated using the results of Step 1.

#### Refined network inference

All elements associated with silent species are removed from the transition matrix 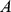 to form the refined transition matrix 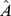. Therefore, the refined system model is as follows: 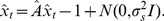
(15)


Although the refined transition matrix 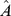 is sparse, it cannot be inferred by Bayesian compressive sensing directly. This is because Bayesian linear regression infers a distribution of 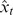 rather than a specific value. If we would like to apply Bayesian compressive sensing to infer the distribution of 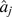, then only the mean of 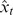 distribution is used for calculation. In this case some information is ignored. Thus, we extend Bayesian compressive sensing to extract information from distributions not just from their mean.

The E-step of the extended Bayesian compressive sensing infers a posterior distribution of 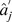, which represents the 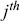 row of 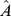, from the posterior distributions of the concentrations 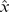. The posterior is a multivariate Gaussian distribution with the mean vector 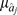 and the covariance matrix 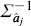 as follows: 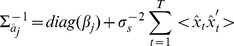
(16)

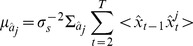
(17)where we have:
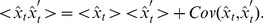
(18)

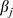
 denotes the hyperparameter of 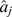. 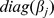 represents a diagonal matrix whose diagonal is vector 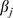. The angled brackets 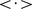 denote the expectation of a distribution. 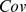 represents the covariance of two random variables.

The M-step of the extended Bayesian Compressive sensing, which is identical to Bayesian compressive sensing, aims to calculate approximately optimal hyperparameters using the variational method [32] as follows: 
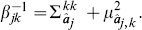
(19)


## Results

A set of benchmark cell-scale networks is designed to demonstrate our method. Each cell-scale network contains 300 species, while only a fraction of species are time-variant over the investigated timescale. The dynamics of these networks are modelled using systems of linear functions. The dimensions of the transition matrices are 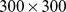. For a time-invariant species 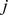, the elements of 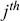 row in the transition matrix are all zero except the 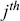 element having the value of 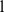; for a time-variant species, its corresponding row has more than one non-zero elements to represent its interactions with other species in the network.

The set of benchmark cell-scale networks has varying numbers of time-variant species. The rows of transition matrices for time-variant species are constructed by taking the structures of 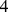 well-studied signalling pathways: JAK-STAT [2], GR [35], ERK [1], p38 [3], and crosstalk amongst them [5], [6], [35]. Details of the benchmark set are listed in Table 1.

**Table 1:** Characteristics of the benchmark network set.

ID	Components	# species	# time-variant species	# links
n-4	JAK-STAT	300	4	4
n-11	ERK	300	11	20
n-39	p38	300	39	61
n-50	ERK and p38	300	50	83
n-53	GR, ERK and p38	300	53	93
n-58	GR, JAK-STAT, ERK and p38	300	58	101

In order to study the effect of perturbations, where various doses or types of inhibitors/stimuli perturb the initial state of the network, different numbers of perturbations are used to simulate benchmark networks. In our simulation, we check the performance of CCELL with the number of perturbations varying from 2 to 7 as these values are frequently used in wet-lab experiments. Under each perturbation, the initial concentration of each species is randomly generated from a normal distribution with mean of 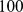, standard deviation of 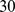. Concentrations of 300 species at 5 sequential time points are generated using benchmark network model and corresponding initial state. For each time point, 150 combined-measurements are carried out according to a predefined measurement matrix. The measurement matrix is generated using low-density parity-check code [36]. Our experiments only focus on investigating the performance of our method when both system noises and measurement noises are maintained at small level (standard deviation 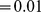). The code and benchmark network set are available at http://dsg.doc.ic.ac.uk/publications/ccell/.

**Figure 3:**
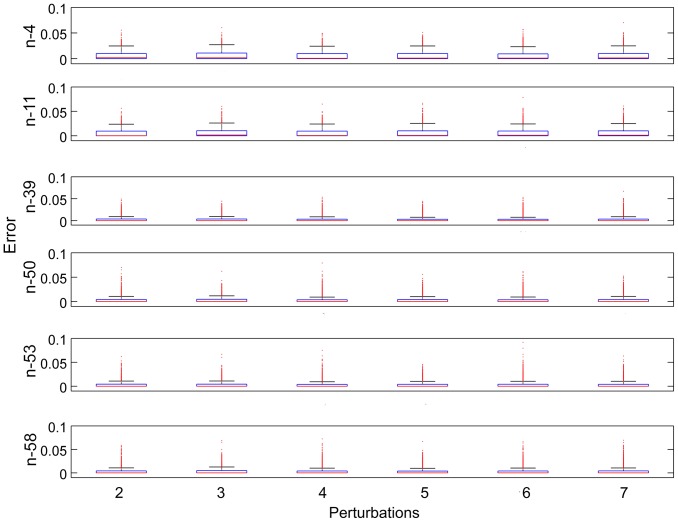
Boxplots of RMSE of inferred concentrations. The 6 subplots depict the results of applying inference method to 6 benchmark networks. For each network, its inference results under different numbers of perturbations, varying from 2 to 7, are shown individually. The median values of RMSE approximate to 0 and the 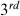 quartile values range from 0.0031 to 0.011.

Figure 3 depicts RMSE values of inferred concentrations for the 6 benchmark networks under 6 different numbers of perturbations. RMSE values in Figure 3 are calculated using differences between inferred concentrations and true concentrations. Almost all RMSE values are below 0.05, except some outliers. Most of the RMSE values are in the range between 0 and 0.011. This indicates that our method accurately and stably infers the concentrations. It can be clearly observed that the RMSE values are not influenced by the number of perturbations, which is consistent with the principle of our method that concentrations of each time point are inferred independently. As can be seen in Figure 3, there are no significant differences between the RMSE values of different benchmark networks. However, the RMSE values of the two networks with high sparsity of variations, n-4 and n-11, are slightly greater than the other three networks. This might be because prior distributions of Bayesian compressive sensing are not sparse enough.

**Figure 4:**
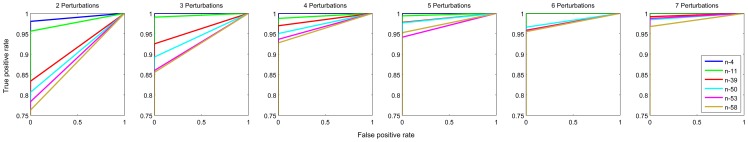
ROC curves of network structure inference. The performance of structure inference, under 6 different numbers of perturbations (from 2 to 7), is evaluated by ROC curves. Each subplot contains the inference results for 6 benchmark networks. The average AUROC is 0.97. More specifically, the maximum AUROC value 1.0 is achieved by the n-4 network (3–7 perturbations) and the n-11 network (6–7 perturbations), while the minimum AUROC value 0.88 is obtained by the n-58 network (2 perturbations).

**Figure 5:**
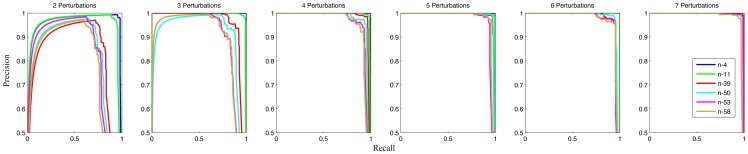
Precision-recall curves of network structure inference. The performance of structure inference, under 6 different numbers of perturbations (from 2 to 7), is evaluated by Precision-recall curves. Each subplot contains the inference results for 6 benchmark networks. The average AUPR is 0.95. More specifically, the maximum AUPR value 1.0 is achieved by the n-4 network (3–7 perturbations) and the n-11 network (6–7 perturbations), while the minimum AUPR value 0.75 is obtained by the n-58 network (2 perturbations).

**Figure 6:**
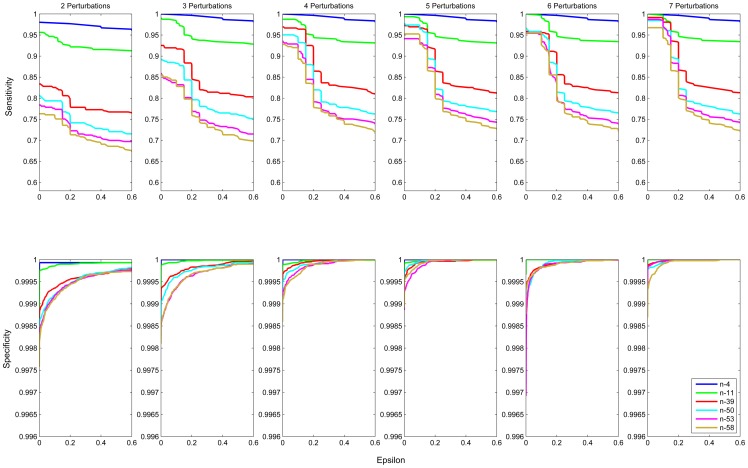
Sensitivity/specificity v.s. threshold parameter. These graphs show the relationships between sensitivity (above) and specificity (below) and threshold parameter 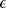 for 6 different benchmark networks with different numbers of perturbations varying from 2 to 7. For 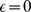, the average specificity is 0.9989 and the average sensitivity reaches its maximum value of 0.9453. When 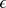 increases to 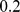, the average specificity is 0.9999 and the average sensitivity decreases to 0.8742. If 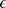 increases to a relatively large value 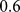, the average specificity achieves 1.000 but the average sensitivity becomes 0.8188.

After obtaining the inferred transition matrix 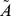 of a network in Step 2, the structure of the network is calculated using structural indicator 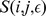 according to Equation 11. A link from species 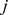 to 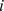 are inferred, if 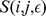 = 1. Varying the threshold parameter 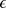 results in different structures. To show the performance of inferring real links in the target networks, ROC and Precesion-recall curves of 6 benchmark networks under 6 different numbers of perturbations are drawn in Figure 4 and Figure 5 respectively. An inferred link is true positive, if it does exist in the network; otherwise, it is false positive. The average of all AUROC and AUPR values is as high as 0.97 and 0.95 respectively, which demonstrates the effectiveness of our method. As evident from Figure 4 and Figure 5, the AUROC and AUPR values rise up as the number of perturbations increases. This indicates that adding new perturbations is an effective way to boost the performance of structure inference. The sparsity of variations is another factor to affect the performance. The AUROC and AUPR values positively correlate with the sparsity of variations. Figure 6 demonstrates relationships between sensitivity or specificity and threshold parameter 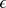 for 6 different benchmark networks with different numbers of perturbations varying from 2 to 7. When 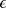 increases from 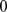 to 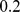, the average sensitivity falls from 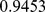 to 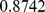 and the average specificity maintains close to 1. The decrease of the average sensitivity is significant, while the change of the average specificity is negligible. The stability of the average specificity is caused by high sparsity of cell-scale signalling networks. Thus, we fix 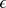 to be 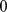 in the following experiments. For simplicity, we suggest that except special conditions users should set 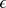 to be 0 for sparse networks.

**Figure 7:**
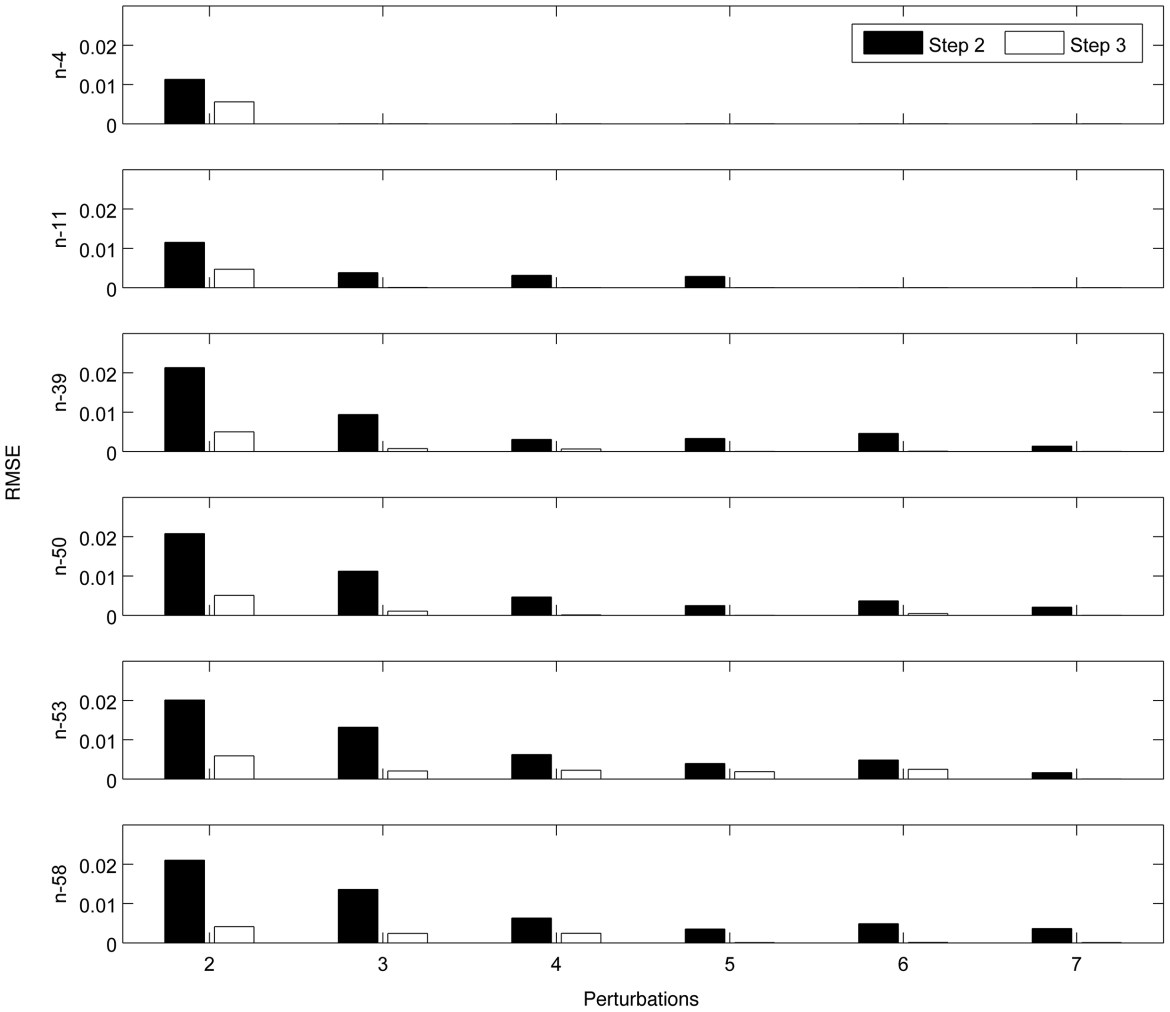
Bar charts of RMSE for inferred transition matrices. These charts show the results of both Step 2 and Step 3 for 6 different benchmark networks with different numbers of perturbations varying from 2 to 7. In Step 2, the RMSE values range from 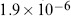 to 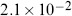 with the mean value of 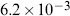; in Step 3, the RMSE values range from 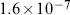 to 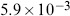 with the mean value of 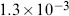. The RMSE ratios (Step 3/Step 2) vary from 0.14% to 51% with the mean value of 17%.

Figure 7 illustrates the RMSE values of transition matrices inferred by both Step 2 and Step 3 for 6 benchmark networks under 6 different numbers of perturbations. RMSE values in Figure 7 are calculated using differences between the elements of the inferred transition matrix and the corresponding elements of true transition matrix. Step 2 infers the transition matrices of a whole network, while Step 3 only infers transition matrices of a refined network only containing active species. In Step 3, the threshold parameter 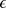 is chosen to be 0. In order to fairly compare the results of Step 2 and Step 3, RMSE values in Figure 7 only calculate the errors in refined transition matrices. Similar to AUROC values, the RMSE value correlates with the number of perturbations and the sparsity of variations. The correlation between the RMSE value and number of perturbations is much stronger than the correlation between the RMSE value and sparsity of variations. It can be seen in Figure 7 thatz the RMSE value decreases significantly to a stable and small value as the number of perturbations increases. The number of perturbations required to reach a stable RMSE value varies across different networks with various sparsity of variations. For the results of Step 3, the n-4 network only needs 3 perturbations, while the n-39 network requires 5 perturbations. It is visible that the convergence rate of results of Step 3 is higher than that of Step 2. What's more, the RMSE values of Step 3 are always smaller than those of Step 2. The RMSE ratios (Step 3/Step 2) vary from 0.14% to 51% with the mean value of 17%, which demonstrates Step 3 substantially improves the performance of transition matrix estimation. It is also clear that Step 3 is more robust than Step 2 under varying number of perturbations.

**Figure 8:**
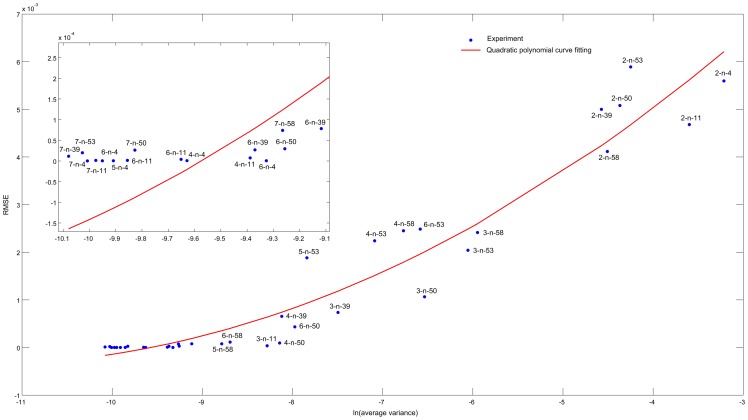
Relationship between the average variance and RMSE. Each point represents an experiment for a benchmark network under a specific number of perturbations. For example, 2-n-53 means the experiment for n-53 network under 2 perturbations. The x-coordinate indicates the natural logarithm of the average variance for all elements in the refined transition matrix, while the y-coordinate indicates the RMSE values of the refined transition matrix. The RMSE values range from 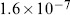 to 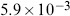 and the average variance varies from 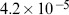 to 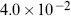.

Figure 8 shows the relationship between the average variance for all elements of the inferred transition matrix according to Equation 16 and their RMSE values. The RMSE values range from 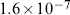 to 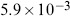, while the average variance varies from 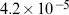 to 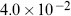. As illustrated by Figure 8, the RMSE value and the average variance have strong correlation that can be well fitted by a quadratic curve, having the Spearman's correlation coefficient to be 0.94. Thus, the average variance is a promising way to represent the accuracy of the inference results when RMSE cannot be calculated due to the unavailability of the real transition matrix. One potential usage of average variance is to adjust the threshold parameter 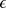. Specifically, when we get different inference results using different 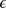, we can choose the most appropriate 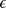 value which results with lowest average variance.

**Figure 9:**
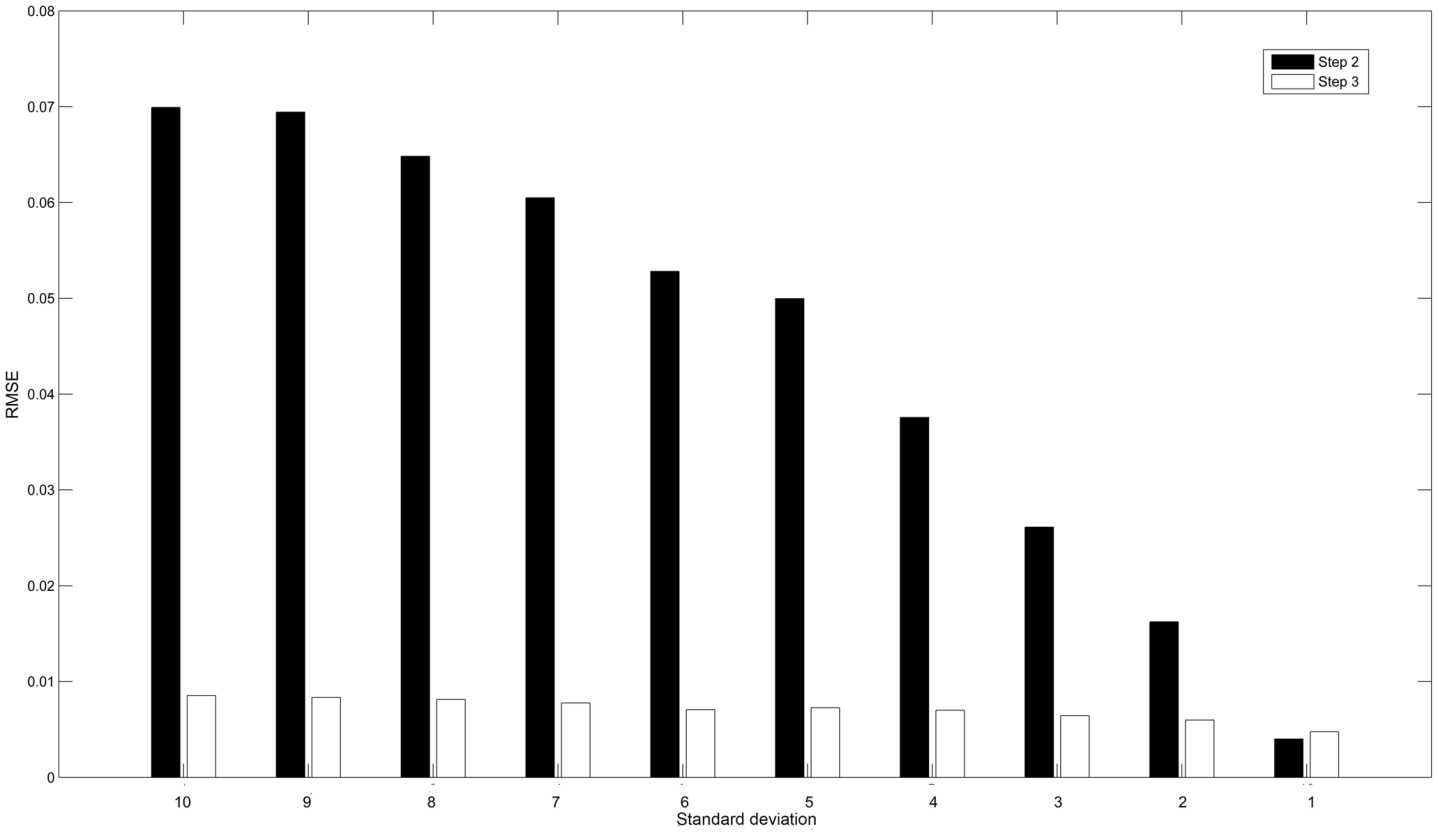
Relationship between noise levels and RMSE. This chart shows the RMSE values for inferred transition matrices of n-39 network under 6 perturbations at different noise levels. The standard deviations of noises vary from 10 to 1. In Step 2, the RMSE values range from 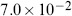 to 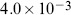; in Step 3, the RMSE values range from 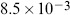 to 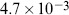.

We stress that the promising results obtained in the above experiments are conditioned on stringent constraints of noises. To investigate the performance of our method in the presence of significant noises, the noises are set to be higher than those in previous experiments. That is, the standard deviations of noises vary from 10 to 1 (signal mean is 100). For n-39 network under 6 perturbations, Figure 9 reveals the relationship between noise levels and the RMSE values of transition matrices inferred by both Step 2 and Step 3. The RMSE values of Step 3 can be always achieved larger or close to those obtained in Step2. The RMSE values of both steps gradually decline with the reduced noise levels. The RMSE values of Step 2 have been decreased by 94%, while the decrease for Step 3 is 44%.

## Discussion

This paper addresses the problem of inferring a cell-scale signalling network as a whole without dividing it into several local networks. We propose a method, which is called CCELL, to solve this problem. The core of this method is Bayesian compressive sensing. To meet the prerequisites of Bayesian compressive sensing, our method is based on two key observations: 1) variations of concentrations are sparse due to separations of timescales; 2) combined-measurements can be implemented using cross-reactivity. To the best of our knowledge, CCELL is the first attempt to infer cell-scale signalling networks from a holistic perspective by exploring separation of timescales and cross-reactivity. We demonstrate that CCELL is effective for inferring benchmark cell-scale networks without structure constraints. Instead of exhaustively measuring all individual species, we show that 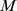 combined measurements are sufficient to infer the network model with acceptable accuracy, where 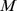 equals to the half of the total number of species in the network.

This paper models biological networks as linear dynamical systems. A classical algorithm to infer the parameters of a linear dynamical system is the expectation maximization (EM) algorithm. The E-step is to infer a distribution of hidden variables (concentrations) using the forward-backward algorithm based on current estimates of parameters. The M-step is to update parameters based on the distribution of hidden variables inferred in the E-step. The E-step and M-step are executed in an iterative way. An advantage of the forward-backward algorithm is that it uses the transition matrix of two adjacent time points of hidden variables to boost the accuracy of hidden variables. However, the transition matrix inferred in M-step is not very accurate, especially when observed data is scarce, while the forward-backward algorithm assumes the transition matrix is highly accurate. This will usually make the EM algorithm overfit for the observed data. Thus, CCELL uses a two-step style rather than an EM style to avoid overfitting.

The measurement matrices in the experiments are generated using low-density parity-check code. In the future, we will study the similarity of all involved proteins, such as their sequence and 3D structures, in order to build a database holding candidates of combined-measurements. All measurements in wet-lab experiments will be selected from this database. This paper focuses on inferring cell-scale signalling networks over a predetermined timescale. By repeating the measurement and inference procedures over different timescales, multiple timescale-specific network models can be obtained. How to integrate them into a unified whole is itself an attractive problem.

CCELL is a promising routine to reveal the mechanism of a complex cellular signal transduction system from a holistic perspective. The current situation, where cell-scale signal transduction models are rarely built due to its difficulty, may be changed. Signalling network databases can be built more efficiently by incorporating much more cell level models to comprehensively understand complex biological processes. Better understanding of complex biological processes is fundamental to understand life and design drugs.
